# Brazilian Red Propolis Extract-Loaded Colloidal Systems Derived from Microemulsion Compositions: Physicochemical, Antioxidant, and Antileishmanial Properties

**DOI:** 10.3390/pharmaceutics18101284

**Published:** 2026-10-10

**Authors:** Salvana P. M. Costa, Ighor C. Barreto, Kathylen V. F. dos Santos, Nataly C. S. Gama, Monique A. Vila Nova, Victor Hugo V. Sarmento, Louisianny G. da Rocha, Irinaldo D. Basílio, Camila M. B. R. G. Panjwanig, Ticiano G. do Nascimento

**Affiliations:** 1 Post Graduate Program in Pharmaceutical Sciences, Institute of Pharmaceutical Science, Federal University of Alagoas, Maceio 57072-970, Alagoas, Brazil; nataly.gama@icf.ufal.br (N.C.S.G.); monique.nova@icf.ufal.br (M.A.V.N.); irinaldo.junior@icf.ufal.br (I.D.B.J.); ticiano@icf.ufal.br (T.G.d.N.); 2 Post Graduate Program in Pharmaceutical Sciences, Department of Life Sciences, State University of Bahia, Av. Silveira Martins, 2555, Cabula, Salvador 41150-000, BA, Brazil; 3 Environmental Coordination Office, Federal University of Bahia, Av. Adhemar de Barros, Ondina, Salvador 40170-110, BA, Brazil; ighor.barreto@ufba.br; 4 Post Graduate Program in Pharmaceutical Sciences, Department of Pharmaceutical Sciences, Federal University of São Paulo, Rua São Nicolau, 20, Centro, Diadema 09913-030, SP, Brazil; kathylen.santos@unifesp.br; 5 Department of Chemistry, Federal University of Sergipe, Avenida Vereador Olímpio Grande, S/N, Professor Alberto Carvalho Campus, Itabaiana 49500-000, SE, Brazil; vhsarmento@gmail.com; 6 Parasitology Laboratory, Center for Biosciences, Federal University of Rio Grande do Norte, Lagoa Nova, Natal 59010-180, RN, Brazil; louisianny@yahoo.com.br; 7 School of Dentistry, Federal University of Alagoas, Av. Lourival Melo Mota, S/N, Tabuleiro do Martins, Maceió 57072-900, AL, Brazil; camila.ribeiro@foufal.ufal.br

**Keywords:** propolis, colloids, surfactant, microemulsions, antioxidant activity, *Leishmania infantum*

## Abstract

**Background/Objectives**: Brazilian red propolis is a rich source of isoflavonoids with promising antioxidant and antileishmanial activities. However, its poor aqueous solubility and chemical instability limit its pharmaceutical application. This study aimed to develop microemulsion-based systems containing high concentrations of Brazilian red propolis extract (RPE) and evaluate their physicochemical properties, phytochemical composition, antioxidant capacity, and antileishmanial activity. **Methods**: Pseudoternary phase diagrams were constructed by water titration to select suitable microemulsion compositions. RPE was subsequently incorporated into the selected compositions to obtain RPE-loaded colloidal systems. The three selected RPE-free microemulsions and their corresponding RPE-loaded systems were characterized by pH, electrical conductivity, dynamic light scattering, rheological measurements, and polarized light microscopy (PLM). The RPE-loaded systems were further evaluated for phenolic marker content, antioxidant activity, and in vitro activity against *Leishmania *infantum. **Results**: Three RPE-loaded colloidal systems (F2-RPE12, F3-RPE30, and F4-RPE50) were developed. F2-RPE12 exhibited characteristics consistent with an oil-in-water microemulsion. Conductivity and PLM indicated physicochemical changes after RPE incorporation in F3-RPE30 and F4-RPE50, with localized birefringent features at higher RPE concentrations. Among the RPE-loaded systems, F4-RPE50 showed the highest phenolic and flavonoid contents and antioxidant activity. The RPE-loaded systems exhibited lower IC_50_ values than RPE alone, with IC_50_ values of 3.14, 2.28, and 0.56 µg·mL^−1^ for F2-RPE12, F3-RPE30, and F4-RPE50, respectively, compared with 5.90 µg·mL^−1^ for RPE. **Conclusions**: Overall, F4-RPE50 showed the most favorable phytochemical and biological performance, and the physicochemical and optical changes observed in the RPE-loaded systems open new avenues for investigating the relationship between structural organization and formulation performance.

## 1. Introduction

Brazilian red propolis (BRP) is an important natural bee product, obtained by *Apis mellifera* L. (Apidae) bees from the resins of the plant species *Dalbergia ecastaphyllum* (L.) Taub. (Fabaceae) and *Symphonia globulifera* L.f. (Clusiaceae). It is enriched in the hive with the bee’s enzyme-rich saliva, wax and pollen [1]. It is widely recognised for its therapeutic potential and agro-industrial applications [2] and it is the second most produced and commercialised type in Brazil [1]. Although it is produced in smaller quantities than green propolis, its market value is approximately twice as high. BRP is mainly produced in the north-east of the country, particularly in the states of Alagoas, Bahia, Sergipe, Pernambuco and Paraíba, where it constitutes a significant commercial asset for local beekeeping [3,4].

In addition to its economic value, BRP is distinguished by its high concentration of isoflavones, flavonoids, chalcones, pterocarpans [5], terpenes, polyprenylated benzophenones (guttiferones), condensed tannins, and phenolic acids [6,7]. Its chemical composition, however, varies according to geographic origin, seasonality and climate, factors that affect standardization and industrial applications [5,8]. This phytochemical diversity gives it a wide range of biological activities, with its antiparasitic action against several species of *Leishmania* standing out in recent years, including *Leishmania infantum* (Trypanosomatidae) [9,10].

Leishmaniasis is a neglected tropical disease. The species *L. infantum* has tropism for organs, primarily the liver, spleen, and bone marrow, causing visceral leishmaniasis (VL), the most severe clinical type, which is almost always fatal if left untreated [11]. More than 90% of VL cases in Latin America occur in Brazil, resulting in a serious public health problem [12]. The pharmacotherapeutic options, amphotericin B, miltefosine, sodium stibogluconate, pentamidine and meglumine antimoniate (Glucantime), are among the few antileishmanial treatments approved by regulatory agencies worldwide [13]. However, these options have limitations that include high toxicity, long treatment periods, development of resistant parasites and high costs [14], which reinforces the need for new therapeutic strategies.

Red propolis emerges as a promising alternative because it contains metabolites capable of interfering with the parasite’s biochemical targets [15]. However, chemical instability in the presence of air, light, and humidity, as well as its immiscibility in water, limit its direct use via oral administration, which reduces its bioavailability and pharmacological activity [16]. Therefore, nanostructured colloidal systems, such as microemulsions (MEs), can be used to overcome these limiting properties of propolis, enhancing its effectiveness [17,18,19].

MEs present several advantages such as thermodynamic stability, dispersion of droplets on a nanometric scale, solubilization of active hydrophobic or hydrophilic compounds in water, such as the constituents of propolis, with the aid of surfactants and/or co-surfactants, which allows the reduction in surface and interfacial tension, keeping the system dispersed [20]. Depending on the composition, pH or temperature, structural transitions may occur, resulting in changes in microstructures or the formation of transition systems [21]. The incorporation of red propolis into these systems may prevent chemical degradation, mask strong taste and odor, and promote the controlled release of bioactive components, improving their efficiency [17,18,19].

Despite the widespread use of microemulsions as delivery systems for poorly soluble bioactive compounds, incorporation of complex natural extracts at high concentrations may substantially modify their physicochemical and colloidal properties. Understanding these changes, together with their possible implications for bioactive compound retention and biological performance, is therefore relevant for the development of colloidal delivery systems containing complex natural products.

The present study investigates the development and characterization of colloidal systems containing increasing concentrations of Brazilian red propolis extract. Their physicochemical properties, optical behavior, phenolic compound content, antioxidant capacity and antileishmanial activity were systematically evaluated, with particular attention to the changes associated with increasing RPE incorporation.

## 2. Materials and Methods

### 2.1. Materials

Red propolis extract (RPE) from Fernão Velho’s Smart Company (Maceió, Brazil) was purchased in 150 mL plastic bottles ready for consumption. Access to the Brazilian genetic heritage associated with the red propolis used in this study was registered in the National System for the Management of Genetic Heritage and Associated Traditional Knowledge (SisGen/CGen, Brazilian Ministry of the Environment) under registration number A827143. Oleic acid, catechin and quercetin dihydrate analytical standards, 2,2-diphenyl-1-picrylhydrazyl (DPPH•), and Folin–Ciocalteu reagent were purchased from Sigma-Aldrich^®^ (São Paulo, Brazil). *Moringa oleifera* oil and *Dipteryx alata* (baru) oil were obtained from Alpha Chemika (Mumbai, India). Analytical-grade ethanol, Tween 20 and Tween 80 were obtained from Oxford Lab Chem (Palghar, India). All other reagents were of analytical grade.

### 2.2. Solubility Assay

The solubility assay was performed to select suitable components for the oil and interfacial phases of the microemulsion systems. RPE solubility was evaluated in each oil, surfactant, and co-surfactant individually, as well as in surfactant/co-surfactant combinations used to compose the interfacial phase. An excess amount of RPE was progressively added to 2 mL of each individual component or surfactant/co-surfactant mixture and mixed at room temperature using a vortex mixer for 30 min to promote solubilization. The samples were then allowed to stand for 5 min to permit the non-solubilized excess to sediment. An aliquot of the resulting liquid phase was collected, and the solubilized RPE was quantified by UV–Vis spectrophotometry using a previously established calibration curve for quercetin as a flavonoid marker. The results were used to compare the RPE-solubilizing capacity of the individual components and surfactant/co-surfactant mixtures and to support the selection of the oil and interfacial phase constituents for microemulsion development. As this assay was performed as a preliminary screening step for component selection, each condition was evaluated using a single preparation and a single determination.

### 2.3. Pseudoternary Phase Diagrams and Formulation Preparation

To determine the concentration ranges at which microemulsions would form, four pseudoternary phase diagrams were constructed using the water titration method [20]. These diagrams used oleic acid as the oil phase and varied the surfactant phase (Tween 20, Tween 80, or a combination of the two) and the co-surfactant (ethanol). Initially, five blends of surfactant and co-surfactant (S/CoS) systems were mixed for 24 h at the following ratios: Tween 20/ethanol 1:1 (*w/w*) (Sm1); Tween 20/ethanol 2:1 (*w/w*) (Sm 2); Tween 20/ethanol 3:1 (*w/w*) (Sm 3); Tween 80/ethanol 2:1 (*w/w*) (Sm 4); and Tween 20:Tween 80:ethanol 3:1:1 (*w/w*) (Sm 5). The oil phase and the S/CoS dispersions were then homogenised in the following proportions: 9:1, 8:2, 7:3, 6:4, 5:5, 4:6, 3:7, 2:8 and 1:9 (*w/w*). This was done using a magnetic stirrer at 100 rpm for 30 min to stabilise the S/CoS systems.

Each mixture was then titrated with distilled water under magnetic stirring for a further 30 min. All formulations were maintained at room temperature for five days before macroscopic and microscopic evaluation. Samples were then observed for macroscopic and microscopic appearance using polarised light microscopy to obtain pseudoternary phase diagrams. Transparent or translucent systems were classified as microemulsions (ME); cloudy liquid systems as coarse or fine emulsions (EM); viscous systems as gels (Gel); and biphasic systems as phase separation (PS). The regions of each type of system were highlighted in pseudoternary phase diagrams using Origin 8 software.

To prepare RPE-loaded colloidal systems, RPE was incorporated at concentrations ranging from 10 to 50% (m/v), calculated relative to the final formulation volume. The RPE was first incorporated into the oil phase, followed by the addition of the surfactant/co-surfactant mixture (S/CoS) under magnetic stirring until complete solubilization. The system was then titrated with distilled water under continuous stirring using the previously described procedure for microemulsions without extract until clear, homogeneous formulations were obtained. The selected RPE-free microemulsions and the corresponding RPE-loaded colloidal systems were maintained at room temperature for 12 months and macroscopically evaluated for phase separation, precipitation, and other visible changes.

### 2.4. Conductivity and pH Characterisations

Electrical conductivity of the formulations was measured at 25 ± 2 °C using a conductometer (Lutron CD-4301), which was calibrated using a KCl standard solution. Before each measurement, the electrode was washed once with distilled water, followed by absolute isopropanol, and then twice with the formulation. Conductivity was expressed in microsiemens per centimeter (µS·cm^−1^). The device was calibrated using 1413 and 12,880 µS·cm^−1^ standards. The pH values of the tested formulations were determined in triplicate using a digital pH meter (Tecnopon mPA 210), which was standardised with pH 4 and pH 7 buffers prior to use.

### 2.5. Dynamic Light Scattering (DLS)

The hydrodynamic diameter and polydispersity index (PDI) of the formulations were measured by dynamic light scattering using a Zetasizer Nano-ZS instrument (Malvern Instruments, Worcestershire, UK), according to the method described by Biswas et al. (2024) [22]. Prior to analysis, the formulations were diluted 1:100 (*v*/*v*) with high-purity water, and the measurements were performed using reusable cuvettes. For each formulation, three independent 1:100 (*v*/*v*) dilutions were prepared from the same sample and analyzed separately (*n* = 3). The results are expressed as mean ± standard deviation (SD).

### 2.6. Polarized Light Microscopy (PLM)

The isotropic behaviour of the formulations was evaluated at room temperature using PLM. A drop of each sample was placed between a coverslip and a glass slide, after which it was examined under cross-polarised light using an Olympus BX-51 optical microscope coupled to an LC Color Evolution digital camera (PL-A662) and image analysis Pixelink Capture software (version 3.2). For each formulation, a single slide was prepared and multiple microscopic fields were examined and recorded to assess the optical characteristics of the sample.

### 2.7. Rheological Characterization

The rheological analysis of the formulations was performed using a Discovery Hybrid Rheometer (DHR-1, TA Instruments, New Castle, DE, USA) as described by Barreto et al. (2022) [20]. A cone and plate device (50 mm diameter, 1° cone angle and 200 µm gap) was used to characterise the rheological behaviour of the formulations. Samples were carefully applied to the lower plate to minimise formulation shearing and were allowed to equilibrate for at least 3 min prior to analysis. A single rheological determination was performed for each formulation at 37 ± 0.2 °C. Steady shear rate sweep experiments (flow tests) were performed with shear rate ($\dot{\gamma }$) in the range of 0–200 s^–1^. The shear rate region used was selected based on the strength of resistance to the applied stresses. The viscosity (η) was determined using the Newtonian model ($\sigma =\;\eta \;\dot{\gamma }$) and TA Instruments TRIOS software (version 5.2; TA Instruments, New Castle, DE, USA).

### 2.8. Total Flavonoid Content (TFC) and Total Phenolic Content (TPC)

Total flavonoid content was determined using the method conducted by Nascimento et al. (2018) [23], which involved monitoring the formation of flavonoid-AlCl_3_ complexes in samples treated with 5% aluminium chloride (AlCl_3_) in methanol using UV–Vis spectroscopy.

Working solutions of the RPE-loaded colloidal systems were prepared in 96% ethanol at pre-established concentrations of 50, 100, 150 and 200 µg·mL^−1^ and sonicated to ensure complete solubilization. An aliquot of the sample was added to a 5 mL volumetric flask, containing 3 mL of methanol and 0.1 mL of aluminium chloride solution (5%). The volumetric flask was completed with methanol to the final volume and stirred for 15 s.

The reaction occurred under light protection for 30 min. Absorbance was measured using a Shimadzu UV–Vis spectrophotometer at a wavelength of 425 nm against a blank containing only methanol and aluminium chloride. The total flavonoid content was calculated using a quercetin calibration curve constructed from seven standard concentrations ranging from 1 to 12 μg·mL^−1^ (Y = 0.0716X − 0.0018; R^2^ = 0.9994) and expressed in terms of milligrams of quercetin per gram of propolis extract (QE/g).

Total phenolic content was determined using the Folin–Ciocalteu reagent following the protocols described by do Nascimento et al. (2018) [23]. Specific aliquots for concentrations of 15, 20, 25, and 30 µg·mL^−1^ were obtained from completely solubilized stock solution of the samples (RPE and RPE-loaded colloidal systems at 1 mg·mL^−1^). To reach each concentration, 250 µL of Folin–Ciocalteu reagent (2M solution) was added to a 5 mL volumetric flask, followed by 3 mL of water, then the samples (75, 100, 125, or 150 µL, respectively) were added immediately afterwards. This mixture was stirred for one minute. Soon after, 600 µL of sodium carbonate solution was added and the mixture was stirred for a further 30 s. Finally, water was added to reach the measurement line. The solutions were then kept for 2 h, protected from light. Absorbance was measured using a Shimadzu UV–VIS spectrophotometer at a wavelength of 750 nm against a blank consisting of Folin–Ciocalteu reagent (FCR) solution, sodium carbonate, and deionised water. A calibration curve was constructed from the analysis of different catechin concentrations, ranging from 2.0 to 9.0 µg·mL^−1^, and their respective absorbance values, based on the following calibration equation: Y = 0.0766x + 0.0566, with R^2^ = 0.9981. The results are expressed in terms of milligrams of Catechin per gram of propolis extract (CE/g). For both TFC and TPC determinations, each concentration of each sample was prepared and analyzed in triplicate.

### 2.9. Identification of RPE Markers Using the UFLC-DAD-UV–Vis Method

Identification and quantification of the marker flavonoids in the RPE and RPE-loaded colloidal systems were performed using a modified version of the method described by Farias Azevedo et al. (2018) [24], using a Shimadzu UFLC-DAD-UV–Vis system.

To identify the flavonoids in the RPE-loaded colloidal systems, an initial ethanol solution of each formulation was prepared to obtain an RPE concentration of 5 mg·mL^−1^. To ensure complete solubilization, RPE and RPE-loaded colloidal system samples were sonicated in an ultrasonic bath for 60 min. Subsequently, a 1 mL aliquot of each stock solution was transferred to a 10 mL volumetric flask and diluted to obtain a final RPE concentration of 500 μg·mL^−1^. The solutions were then filtered through a 0.22 µm filter, and analyzed once by injecting 2 µL into the UFLC-DAD-UV–Vis system.

The UFLC-DAD-UV–Vis system comprised the following modules: a high-pressure pump (LC-20ADXR model), a degasser (DGU-20A3R model), an auto-injector (SIL-20AXR model), a chromatographic column oven, diode array detectors (SPD-M20A model) and a fluorescence detector (RF-20A model), a controller (CBM-20A model) and Shimadzu LabSolutions LC software (Shimadzu Corporation, Kyoto, Japan). The flavonoids were separated using a reverse-phase C18 column (150 × 4.6 mm, 5 µm) with a mobile phase consisting of solvents A (reverse-osmosis water) and B (HPLC-grade acetonitrile), pumped at a flow rate of 0.3 mL min^−1^. The initial elution gradient consisted of 70% water and 30% acetonitrile, with the percentage of B increasing to 100% over 40 min. This was followed by an isocratic condition of solvent B (100%) for 3 min, returning to the initial conditions at 54 min, and then an isocratic condition of solvent B (30%) for 6 min. Analytical standards of the described marker flavonoids (daidzein, liquiritigenin, pinobanksin, isoliquiritigenin, formononetin, pinocembrin and biochanin A) were used to identify the flavonoids in RPE and RPE-loaded colloidal systems by comparing ultraviolet spectra obtained using a photodiode array detector at different wavelengths (249, 281, 286, 275 and 366 nm), as well as the retention times obtained for the standards and samples.

### 2.10. DPPH• Radical Scavenging Capacity Method

The DPPH• radical scavenging capacity was measured according to the methodology described by do Nascimento et al. (2018) [23] to evaluate the antioxidant activity of RPE and the RPE-loaded colloidal systems. DPPH is a stable free radical that accepts an electron or a hydrogen radical from the phenolic compounds present in RPE and RPE-loaded colloidal systems. A DPPH· reagent solution was prepared at a concentration of 0.04 mg·mL^−1^ using absolute ethanol. This solution was kept refrigerated and protected from light for up to five working days.

Stock solutions of RPE and RPE-loaded colloidal systems at a concentration of 1 mg·mL^−1^ were then prepared in ethanol. To determine antioxidant activity, 50 μL, 100 μL, 150 μL, 200 μL and 250 μL of the stock solutions were transferred into 5 mL volumetric flasks in triplicate for each concentration and sample. Two millilitres of the DPPH· solution were added to each flask and the flasks were filled with absolute ethanol up to the mark. The concentrations obtained were 10, 20, 30, 40 and 50 μg·mL^−1^. The reaction was initiated and the volumetric flasks were stored in the dark for 30 min. After the reaction had finished, the DPPH decay was analysed using a Shimadzu UV Mini-1240 UV–Vis spectrophotometer in photometry mode with a wavelength set to 518 nm. The percentage inhibition of the DPPH radical by the samples was calculated according to the following equation:

(1) $$\%\;\mathrm{Antioxidant}\;\mathrm{Activity}=\frac{\left(100-((\mathrm{Abs}\;\mathrm{sample}-\mathrm{Abs}\;\mathrm{blank}\right)\;\times \;100)}{\left(\mathrm{Abs}\;\mathrm{control}\right)}$$

where Abs sample = absorbance of RPE or RPE-loaded colloidal systems subjected to the DPPH· radical capture reaction, Abs blank = absorbance of the sample solution without the addition of DPPH· radical, Abs control = absorbance of the DPPH reference solution before the test (DPPH reagent solution).

### 2.11. Antileishmanial Activity

The *Leishmania* (L.) *infantum* BR2000 promastigote strain was donated by the Aggeu Magalhães Institute (Fiocruz, Pernambuco, Brazil) [25]. The promastigote forms were cultured in vitro in Grace’s Insect Medium (Sigma–Aldrich, St. Louis, MO, USA), supplemented with 2 mM L-Glutamine (Gibco BRL, Grand Island, NY, USA), 20% FBS, 5% urea, 50 U mL^−1^ penicillin G potassic (USB Corporation, Cleveland, OH, USA) and 50 µg mL^−1^ streptomycin, with the pH adjusted to 6.5. The culture was then incubated in a BOD incubator at 25 °C.

Sample preparation: RPE and RPE-loaded colloidal systems were weighed accurately and solubilised with DMSO/H_2_O (Milli-Q water) (05/95, V/V) to obtain a stock solution of 3000 µg·mL^−1^. Using this solvent system, the RPE was completely solubilised, whereas the RPE-loaded colloidal systems were only partially solubilised. Several working solutions were obtained by transferring aliquots of the RPE or RPE-loaded colloidal systems stock solutions and diluting them with Schneider’s medium supplemented with 10% FBS, to obtain volumes of 1 mL at concentrations ranging from 75 to 1.5 µg·mL^−1^. This ensured that the concentration of the DMSO solvent did not exceed 0.5%, at which concentration it had no detrimental effect on the parasites [26].

Aliquots (100 μL) of cultured procyclic promastigote cells (at a concentration of 4 × 10^6^ cells·mL^−1^) were immersed in Schneider’s medium containing 10% FBS in 96-well microplates. Then, 100 μL of samples (RPE and RPE-loaded colloidal systems at a concentration range of 75–1.5 µg·mL^−1^) were added to obtain concentrations of 37.5–0.75 µg·mL^−1^, with each concentration tested in triplicate wells on the same plate. The samples were then incubated for 24 h in a BOD incubator at 26 °C. The morphology and viability of the parasites were then evaluated using an inverted microscope (Olympus IX70, Olympus Corporation, Tokyo, Japan) for all concentrations tested. Finally, the number of parasites was determined using an optical microscope with a ×40 objective and a Neubauer chamber to calculate the antileishmanial effect. The results were expressed as the concentration required to inhibit parasite growth by 50% (IC_50_). The positive control used was the drug Amphotericin B in Liposomal form (AmBisome^®^ from GILEAD Sciences) diluted in sterile double-distilled water at concentrations ranging from 0.006 µg·mL^−1^ to 0.030 µg·mL^−1^ (6 to 30 ng·mL^−1^), serving as an internal quality control and a benchmark for comparative potency. The antileishmanial assay was performed according to Rocha et al. (2009) [27].

### 2.12. Statistical Analyses

Data from DLS, total phenolic content (TPC), and total flavonoid content (TFC) analyses are expressed as mean ± standard deviation (SD). TPC and TFC data were analyzed by one-way analysis of variance (ANOVA) followed by Bonferroni’s post hoc test, with statistical significance set at *p* < 0.05. For the DPPH• assay, IC_50_ values were estimated by nonlinear regression and are reported with 95% confidence intervals. IC_50_ values for antileishmanial activity were estimated by nonlinear regression using GraphPad Prism version 5.0. The number and type of experimental determinations or replicates used for each analysis are specified in the corresponding methodological subsections.

### 2.13. Generative Artificial Intelligence-Assisted Illustration

**Figure 6:**
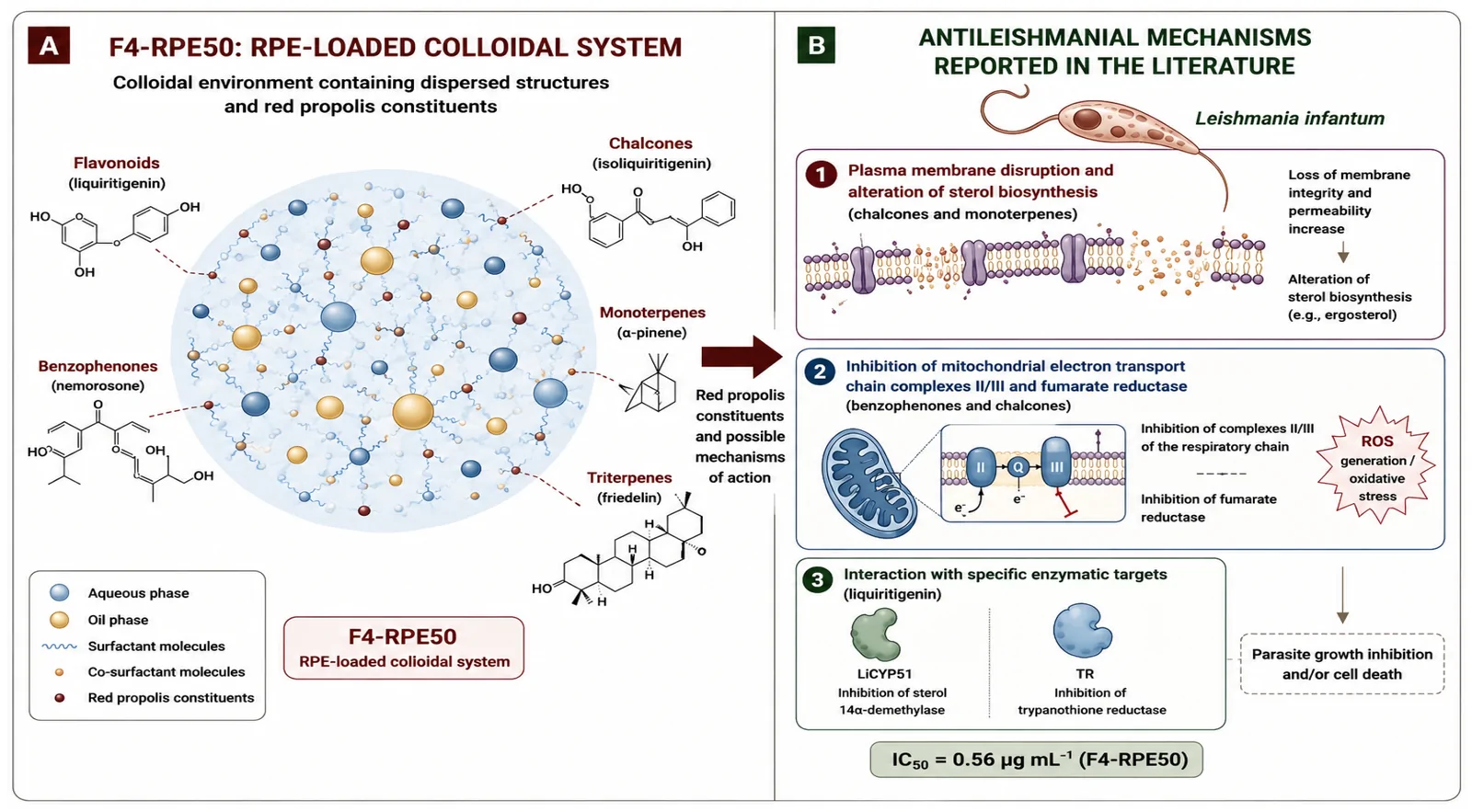
Schematic representation of (**A**) the F4-RPE50 RPE-loaded colloidal system and representative bioactive constituents identified or reported in Brazilian red propolis, and (**B**) antileishmanial mechanisms reported in the literature for red propolis constituents, including: (i) disruption of the plasma membrane and alterations in sterol biosynthesis associated with chalcones and monoterpenes; (ii) inhibition of complexes II/III of the mitochondrial respiratory chain associated with benzophenones and inhibition of fumarate reductase associated with chalcones; and (iii) interaction of liquiritigenin with specific enzymatic targets, including LiCYP51 and trypanothione reductase (TR). Increased ROS generation and oxidative stress have also been associated with mitochondrial effects of some red propolis constituents. The schematic representation does not imply a specific internal microstructure or spatial distribution of the bioactive compounds within F4-RPE50. The antileishmanial mechanisms depicted are based on the previous literature and were not experimentally investigated in the present study.

OpenAI ChatGPT (image generation feature; OpenAI, San Francisco, CA, USA) , with image generation capabilities, was used solely to generate the initial illustrations for Figure 6 and the Graphical Abstract. The AI-generated outputs were subsequently reviewed and edited by the authors to ensure scientific accuracy and consistency with the experimental findings and concepts presented in the manuscript. Generative AI was not used for data generation, data analysis, or interpretation of the results.

## 3. Results and Discussion

### 3.1. Solubility Test for Oil Phase, Surfactant and Co-Surfactant

Microemulsions are primarily composed of an oil phase, surfactant, and co-surfactant. The selection of these components plays a critical role in formulation development, as it influences the amount of active compounds that can be solubilized in the system [28]. Vegetable oils contain high levels of unsaturated fatty acid glyceride esters [20] and are generally considered safe, commonly present in the diet, and readily digestible [29]. Polysorbates (Tweens) are widely used non-ionic surfactants in microemulsion-based drug delivery systems [30,31] due to their compatibility in mixtures [31] and their ability to maintain the stability of emulsified systems over a broad range of pH and electrolyte concentrations [32].

Accordingly, the solubility of RPE was quantitatively evaluated in the individual oil-phase and interfacial-phase candidates, as well as in different S/CoS combinations, as described in Section 2.2. The results are presented in Table 1.

Among the oil-phase candidates, oleic acid showed the highest RPE solubility (7.67 μg·mL^−1^), compared with *Moringa oleifera* oil (4.09 μg·mL^−1^) and baru oil (3.21 μg·mL^−1^) (Table 1). Oleic acid is a biocompatible long-chain fatty acid commonly associated with microemulsion systems [33,34]. In addition to acting as an oil phase, it may contribute to reducing interfacial tension. Oleic acid is also recognized as a penetration enhancer, with previous studies reporting effects on lipid packing and membrane fluidity in biological barriers [33,35].

Among the surfactants evaluated individually, Tween 80 showed higher RPE solubility (9.93 μg·mL^−1^) than Tween 20 (6.64 μg·mL^−1^), whereas ethanol, evaluated as a co-surfactant, showed an RPE solubility of 5.37 μg·mL^−1^ (Table 1).

The solubilizing capacity of different S/CoS combinations was subsequently evaluated. Among the systems presented in Table 1, Tween 20/ethanol at a 3:1 (*w/w*) ratio (Sm3) showed the highest RPE solubility (16.72 μg·mL^−1^), compared with Tween 20/ethanol at 1:1 (Sm1; 9.02 μg·mL^−1^) and 2:1 (Sm2; 8.55 μg·mL^−1^), Tween 80/ethanol at 2:1 (Sm4; 7.90 μg·mL^−1^), and Tween 20/Tween 80/ethanol at 3:1:1 (Sm5; 9.66 μg·mL^−1^). Thus, increasing the proportion of Tween 20 relative to ethanol to a 3:1 ratio resulted in the highest RPE-solubilizing capacity among the evaluated interfacial mixtures.

Considering both RPE solubilization and the intended oral application, systems with lower ethanol content were prioritized. The Tween 20/ethanol 1:1 (*w/w*) ratio was therefore not selected for further development because of its higher relative ethanol content, since ethanol-containing oral formulations may be unsuitable for pediatric patients and other populations for whom alcohol intake should be limited. Based on the overall solubility results and formulation considerations, oleic acid was selected as the oil phase, while Tween 20 and Tween 80 were selected as surfactants for subsequent microemulsion development.

### 3.2. Pseudo-Ternary Phase Diagrams and Design of the Microemulsion System

**Figure 1:**
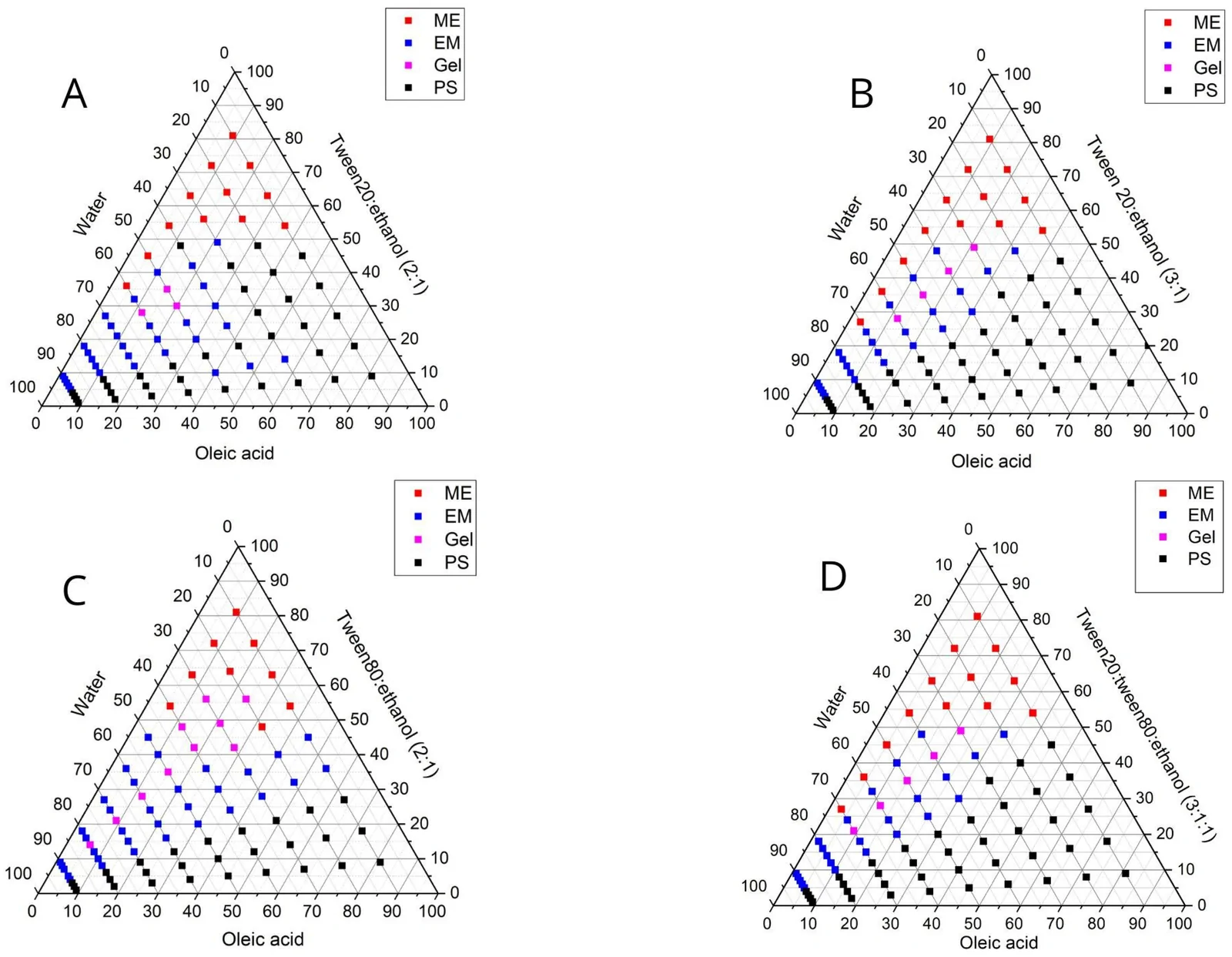
The pseudoternary phase diagram illustrates microemulsion formation at various S-CoS system ratios. (**A**) Tween 20/ethanol, 2:1 *w/w*; (**B**) Tween 20/ethanol, 3:1 *w/w*; (**C**) Tween 80/ethanol, 2:1 *w/w* and (**D**) Tween 20/Tween 80/ethanol ratio 3:1:1 *w/w*/*w*. Red dots indicate the formed microemulsions.

The four phase diagrams constructed are shown in Figure 1.

The different phase regions, i.e., microemulsion (ME), phase separation (PS), emulsion (EM), and gel, are clearly defined. In all diagrams, regions characterized by higher water content and lower S/CoS proportions showed a tendency toward emulsion formation and phase separation. Conversely, the ME regions expanded with increasing surfactant proportions, with water incorporation reaching 50% in Figure 1D, 60% in Figure 1A, and 70% in Figure 1B,C.

As illustrated in Figure 1A,B,D, systems containing Tween 20 exhibited expanded ME regions and greater water incorporation. Similar behavior has been reported by Hathout et al. (2010) [36]. This effect may be attributed to Tween 20’s shorter alkyl chain length, higher hydrophilicity (HLB = 16.7), and greater ability to incorporate water [37]. The presence of ethanol increases the effective HLB of the system and enhances the affinity of nonionic surfactants for the aqueous phase [38].

In contrast, the system containing Tween 80 (Figure 1C) exhibited a limited ME region, with clear phases observed only at low oil-to-Sm ratios (1:9 and 2:8). Much of this diagram was characterized by viscous gel phases at intermediate water contents (20–80%), which subsequently dispersed into coarse, highly viscous emulsions upon dilution. The formation of gel or liquid crystalline phases in Tween 80- based systems has been widely reported and is associated with reduced interfacial flexibility and restricted self-emulsification capacity [39,40].

According to Li et al. (2016) [41], a larger microemulsion region in pseudoternary phase diagrams indicates improved self-emulsifying performance. Among the Tween 20/ethanol systems, increasing the Tween 20-to-ethanol ratio from 1:1 and 2:1 to 3:1 resulted in a slightly expanded ME region (Figure 1B). However, the Sm2 diagram (Tween 20/ethanol, 2:1, *w/w*; Figure 1A) presented a comparable number of transparent ME points (approximately 12) while requiring a lower total surfactant content. This balance between an adequately extended ME region and reduced surfactant concentration is advantageous from formulation and safety perspectives. Therefore, Sm2 (Tween 20/ethanol, 2:1, *w/w*) was selected as the most suitable system for developing microemulsions incorporating Brazilian red propolis extract.

### 3.3. Selection of Microemulsion Compositions and Development of RPE-Loaded Colloidal Systems

Three compositions were chosen from the Sm2 pseudoternary phase, with different proportions of water, surfactant system and oil phase, in order to evaluate their ability to support different RPE loads. The composition of these microemulsions (MEs) is presented in Table 2. RPE was incorporated into formulation F2 at a concentration of 12% (m/v), resulting in formulation F2-RPE12. RPE was also incorporated into F3 and F4 at concentrations of 30% and 50% (m/v) respectively, resulting in F3-RPE30 and F4-RPE50. Following RPE incorporation, the formulations remained visually homogeneous and optically transparent, with no marked macroscopic changes. Therefore, the RPE-free microemulsions and the corresponding RPE-loaded colloidal systems were further characterized. During the subsequent 12-month observation period, the selected RPE-free microemulsions and the corresponding RPE-loaded colloidal systems showed no macroscopic signs of phase separation, precipitation, or other visible changes.

### 3.4. Physicochemical Characterization of Microemulsions and RPE-Loaded Colloidal Systems

#### 3.4.1. Polarized Light Microscopy (PLM)

**Figure 2:**
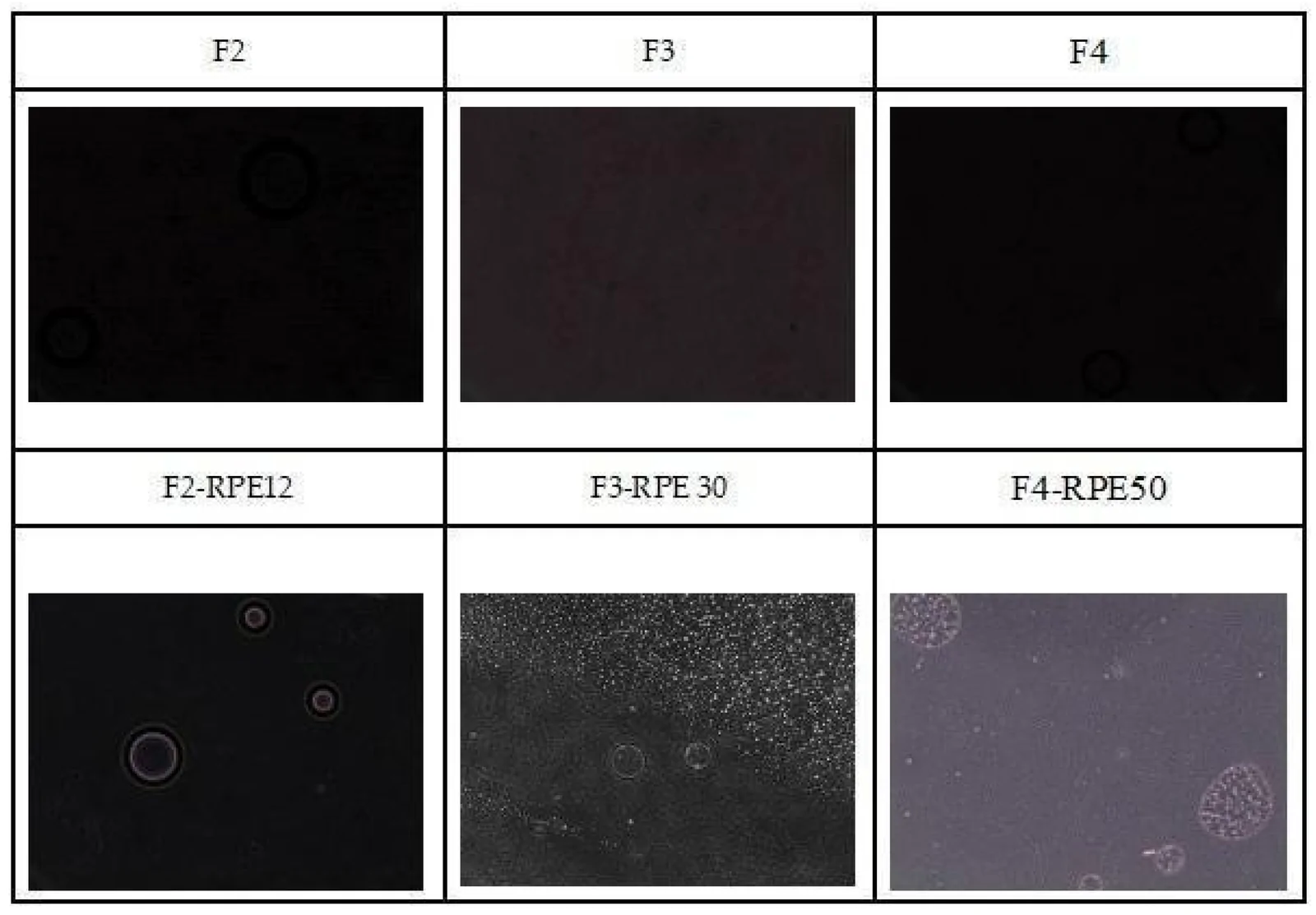
Polarized light microscopy analysis of the RPE-free microemulsions and the corresponding RPE-loaded colloidal systems, acquired using a 20× objective.

Analysis of the RPE-free microemulsions (F2, F3 and F4), as well as F2-RPE12, by PLM did not show detectable birefringence under crossed polarizers, consistent with the optical isotropy of these samples. Optical isotropy is a characteristic commonly associated with microemulsion systems [20,42,43]. Accordingly, optically isotropic samples appear as a dark field under crossed polarizers, whereas anisotropic materials may exhibit birefringent features [44,45], as illustrated in the photomicrographs (Figure 2).

In contrast, F3-RPE30 exhibited an overall dark background with localized birefringent features lacking a characteristic texture, indicating localized optical anisotropy. These features suggest that the optical properties of the system became spatially heterogeneous after RPE incorporation. Similar birefringent behavior has been reported during transitions from microemulsion to liquid-crystalline phases in colloidal systems [34]. The coexistence of isotropic and liquid-crystalline regions has also been described in related systems [46,47]. However, these literature comparisons do not establish the presence of a liquid-crystalline mesophase in F3-RPE30. In turn, F4-RPE50 exhibited larger dispersed structures associated with distinct bright features under crossed polarizers, demonstrating localized birefringence. The surrounding background remained predominantly dark and homogeneous, indicating that optically isotropic regions coexist with the localized birefringent features. However, PLM does not provide sufficient spatial or structural resolution to determine the precise localization or structural nature of these birefringent features.

#### 3.4.2. Dynamic Light Scattering (DLS)

The hydrodynamic diameter ranged from 23.88 to 74.65 nm for extract-free MEs (F2, F3, and F4) (Table 3), which is within the conventional size range reported for microemulsions (5–200 nm) [48]. The PDI of these samples ranged from 0.374 to 0.461.

PDI is a measure of particle homogeneity, and a lower PDI value has been associated with a monodisperse system, with high homogeneity in the droplet population, while higher values indicate a broader size distribution [20,49,50,51]. Particle size and PDI are relevant physicochemical parameters for characterizing microemulsion systems [52].

**Figure 3:**
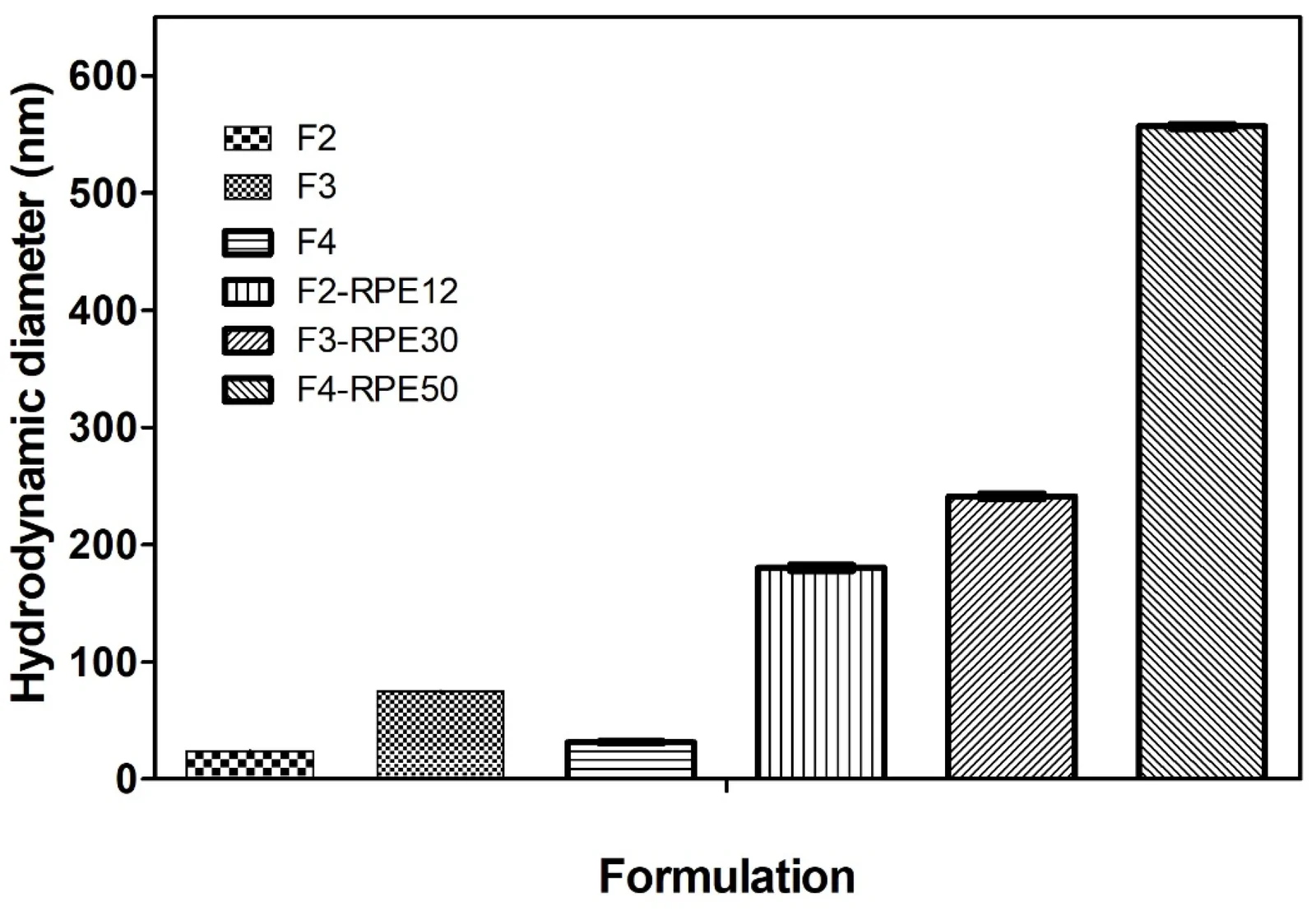
Mean hydrodynamic diameter (Z-average) of the RPE-free microemulsions (F2, F3, and F4) and the corresponding RPE-loaded colloidal systems (F2-RPE12, F3-RPE30, and F4-RPE50), determined by dynamic light scattering (DLS). Data are expressed as mean ± SD of three independently prepared dilutions from the same sample (*n* = 3).

For the RPE-loaded colloidal systems (F2-RPE12, F3-RPE30 and F4-RPE50), the hydrodynamic diameter ranged from 180 to 557 nm, and the PDI ranged from 0.190 to 0.279. The mean hydrodynamic diameters of the RPE-free microemulsions and their corresponding RPE-loaded systems are comparatively illustrated in Figure 3.

Therefore, the broad overall range of hydrodynamic diameters observed across the investigated formulations should not be interpreted as a broad size distribution within a single microemulsion, but rather reflects differences among distinct systems, particularly between the RPE-free microemulsions and their corresponding RPE-loaded formulations. For F2-RPE12, the hydrodynamic diameter increased from 23.88 to 180 nm, while the PDI decreased from 0.461 to 0.190. These characteristics remained compatible with a microemulsion. For F3-RPE30, the hydrodynamic diameter increased from 74.65 to 241.3 nm, exceeding the conventional size range considered for microemulsions, while the PDI decreased from 0.445 to 0.279. For F4-RPE50, a marked increase in hydrodynamic diameter was observed, from 31.35 to 557 nm, while the PDI decreased from 0.374 to 0.223.

These changes indicate that F3-RPE30 and, particularly, F4-RPE50 no longer exhibit the dimensional characteristics typically associated with classical microemulsions. Notably, the marked increase in hydrodynamic diameter observed for F4-RPE50 occurred concomitantly with the incorporation of a higher RPE concentration and with the optical changes detected by PLM. Although hydrodynamic diameter increased after RPE incorporation, particularly for F4-RPE50, the measured values remained within the submicrometric range, consistent with their description as colloidal systems. In addition, the decrease in PDI observed after RPE incorporation indicates a narrower size distribution in the RPE-loaded colloidal systems. The increase in hydrodynamic diameter observed after RPE incorporation may therefore be associated with physicochemical changes accompanying the incorporation of high concentrations of the complex propolis extract into the original microemulsion compositions. A similar trend was reported by Žilius et al. 2016 [53] for propolis-containing microemulsions, in which the incorporation of ethanolic propolis extract was accompanied by an increase in mean droplet size and a decrease in PDI.

#### 3.4.3. Conductivity and pH Characterisations

Electrical conductivity analysis is a useful complementary tool for investigating microstructural changes in self-assembled colloidal systems. Microemulsions may exhibit hydrophobic aggregates in water (o/w), hydrophilic aggregates in oil (w/o), and bicontinuous organization, and variations in conductivity have been associated with changes in aqueous-domain connectivity and structural organization [54,55,56].

Sample F4, with a water mass fraction (Φ_w) of 10% *w/w*, exhibited low conductivity (12.61 µS·cm^−1^) (see Table 2), consistent with a predominantly non-aqueous continuous phase and therefore suggestive of a W/O microemulsion. When the weight fraction of the aqueous phase is low, water droplets are typically dispersed as discrete particles with minimal interaction in the continuous oily medium, resulting in low conductivity [56].

Sample F3, with a water mass fraction of 20% *w/w*, showed an intermediate conductivity of 22.19 µS·cm^−1^. Compared with F4, this increase in conductivity suggests greater electrical connectivity within the system as the aqueous fraction increases. Similar composition-dependent increases in conductivity have been associated with changes in domain connectivity and structural organization in microemulsion systems [56].

Sample F2, with a water mass fraction of 50% *w/w*, showed high conductivity (92.64 µS·cm^−1^), consistent with a predominantly aqueous continuous phase and therefore with an O/W organization. Composition-dependent changes in conductivity have been associated with percolation phenomena in microemulsion systems, particularly within aqueous phase ranges of 20–35% and 50–70% [55,57,58]. According to this model, as the volume fraction of water increases, some of the conductive droplets begin to come into contact with each other, forming clusters that are sufficiently close to increase conductivity [58]. Once a critical water volume fraction is reached and the percolation threshold (Φp) is exceeded, the number of these clusters increases rapidly, resulting in a sharp rise in conductivity [56].

Adding 12% RPE (F2-RPE12) markedly increased conductivity (from 92.64 to 259 µS·cm^−1^), remaining consistent with a predominantly aqueous continuous phase and with the O/W organization proposed for this formulation. Adding 30% RPE (F3-RPE30) increased conductivity from 22.19 to 71 µS·cm^−1^, whereas incorporation of 50% RPE (F4-RPE50) increased conductivity from 12.61 to 28 µS·cm^−1^. These increases indicate enhanced electrical transport within the systems, potentially associated with changes in the connectivity of polar domains and/or the contribution of ionizable components present in RPE. RPE is an ethanolic extract rich in polyphenols, flavonoids, and phenolic acids, whose polar and ionizable constituents may interact with the colloidal domains and modify the physicochemical environment of the systems [59]. The conductivity changes occurred concomitantly with the optical changes observed by PLM.

Adding RPE reduced the pH in both F3-RPE30 (from 5.09 to 4.34) and F4-RPE50 (from 5.42 to 4.10), which may have contributed to the physicochemical changes observed in these systems. Phenolic compounds and acids present in RPE may contribute to the pH decrease and interact with components at the oil/water interface through hydrogen bonding, hydrophobic interactions, and other intermolecular interactions, thereby affecting the physicochemical properties of the colloidal systems [60]. Furthermore, oleic acid, a key component of the interface, is a weak fatty acid with a pKa of approximately 4.8–5.0, and its ionization state is therefore pH-dependent. At pH > 5, oleic acid is partially ionized (R–COO^−^), which can increase the negative charge at the interface and electrostatic repulsion [61,62]. At pH values below its pKa, as observed after incorporation of 30% and 50% RPE, a greater proportion of oleic acid is expected to remain in the protonated form (R–COOH), which is less water-soluble and provides lower electrostatic repulsion. Accordingly, the observed physicochemical changes are consistent with possible alterations in interfacial organization associated with the pH-dependent ionization state of oleic acid, which may have contributed to the increased hydrodynamic diameters observed for F3-RPE30 and F4-RPE50 [63,64,65,66].

The pH of F2 was already 3.88 before RPE incorporation and changed only slightly after addition of 12% RPE (F2-RPE12: 3.99). Therefore, pH variation alone is unlikely to account for the physicochemical changes observed after RPE incorporation. The chemical constituents introduced by RPE may also contribute through interactions with the colloidal components, thereby modifying the physicochemical environment of the system. The increase in conductivity observed after RPE incorporation may therefore reflect the combined influence of system composition and changes in the electrical connectivity of conductive domains, rather than a pH-dependent effect alone.

The acidic pH of F2 prior to RPE incorporation may be related to the composition of the formulation. In particular, oxidative degradation of polysorbates has been reported to generate short-chain organic acids that can contribute to pH reduction in unbuffered formulations [67]. In addition, the higher water content and lower surfactant concentration of F2 may influence the partitioning of acidic species within the system, potentially increasing their presence in the aqueous phase and contributing to the lower pH.

#### 3.4.4. Rheological Measurements

**Figure 4:**
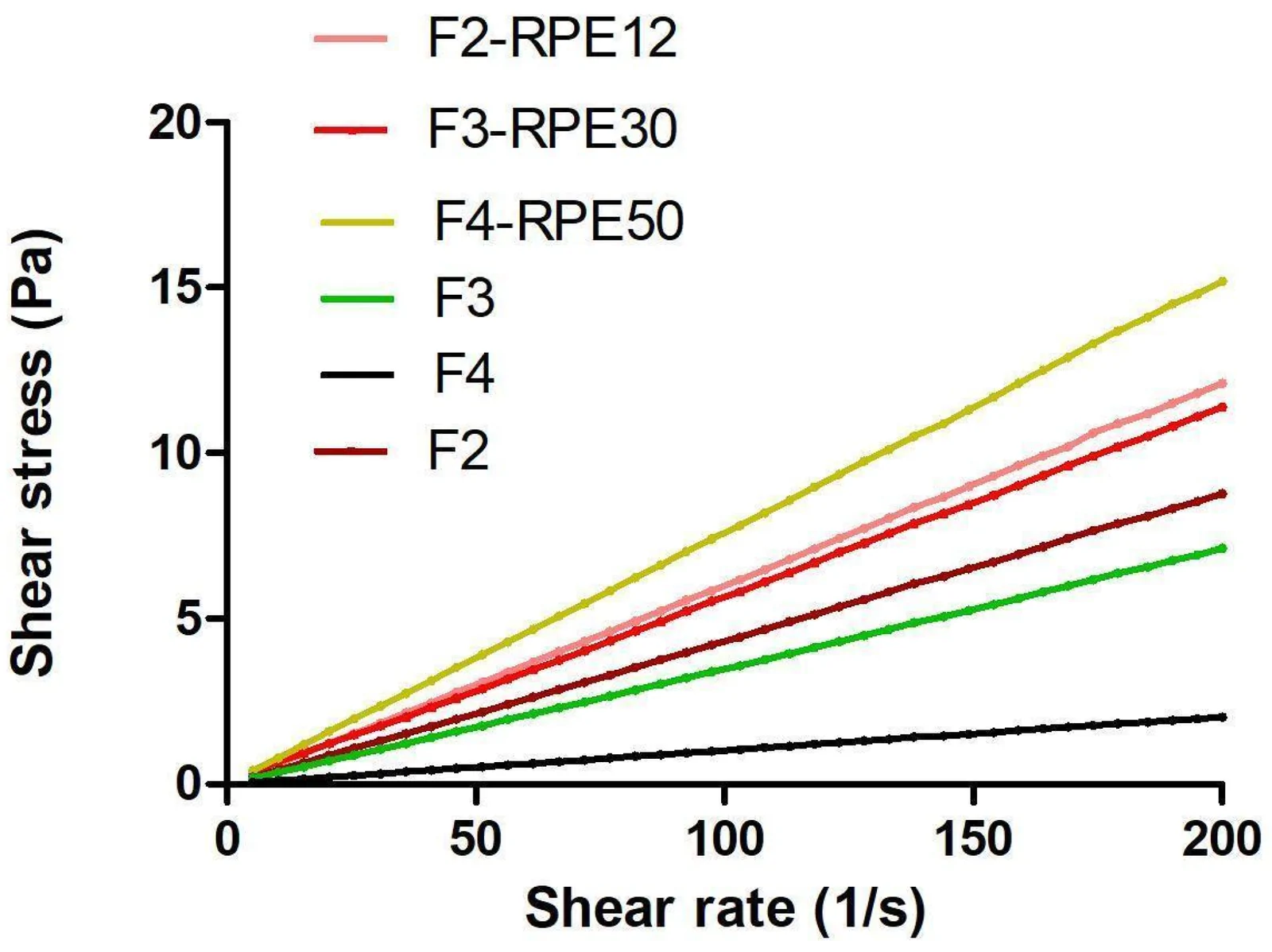
Rheograms of the selected RPE-free microemulsions (F2, F3, and F4) and the RPE-loaded colloidal systems (F2-RPE12, F3-RPE30, and F4-RPE50).

Rheological analysis was performed to determine the viscosity and flow behavior of the formulations under shear. The flow curves showed an approximately linear relationship between shear stress and shear rate (Figure 4), consistent with Newtonian behavior under the experimental conditions employed [20]. The viscosity (η) of the formulations was determined using the Newtonian model, which provided high coefficients of determination (R^2^ > 0.99; Table 3), supporting an essentially Newtonian macroscopic flow response over the experimentally investigated shear-rate range. Newtonian behavior is commonly reported for microemulsion systems and has been associated with the small size of dispersed droplets and relatively limited interactions between them [20,68].

For extract-free samples, viscosity increased with increasing water content. F4 (Φw = 10% *w/w*) exhibited the lowest viscosity, a finding that, together with its low electrical conductivity, is consistent with the proposed W/O organization. Such low-viscosity behavior has been associated with systems characterized by relatively simple dispersion and limited interactions between dispersed aqueous domains [69]. F3 exhibited higher viscosity than F4, indicating that increasing the aqueous fraction from 10% to 20% substantially altered the flow resistance of the system. Increased resistance to flow has been associated with greater interactions and connectivity between domains in colloidal systems [70]. Therefore, the higher viscosity of F3 may reflect composition-dependent changes in colloidal interactions and organization.

Although F2 (Φw = 50% *w/w*) exhibited characteristics consistent with an O/W microemulsion, including high electrical conductivity (92.64 µS·cm^−1^) consistent with a predominantly aqueous continuous phase, its viscosity (43.9 mPa.s) was higher than typically expected for a conventional O/W microemulsion with a comparable droplet size and phase ratio. Percolation phenomena and composition-driven transitions between bicontinuous and O/W organizations have been associated in microemulsion systems with increases in viscosity [71]. In ternary or pseudoternary systems containing surfactants and non-ionic co-surfactants (such as Tween 20 and ethanol), such transitions have been associated with progressive changes in domain connectivity, which may increase resistance to flow while preserving macroscopic optical isotropy [70,72]. The concomitant increase in electrical conductivity and viscosity is consistent with trends reported during composition-dependent transitions in microemulsion systems, including transitions from W/O or bicontinuous organizations toward O/W systems [73].

For RPE-loaded colloidal systems, F2-RPE12, which retained characteristics consistent with an O/W microemulsion, showed increased viscosity after incorporation of 12% RPE, which may be related to the resinous constituents introduced by the extract. Its high conductivity remained consistent with a predominantly aqueous continuous phase and therefore with an O/W organization. The hydrodynamic diameter, low PDI, and Newtonian flow behavior further suggest that incorporation of 12% RPE was compatible with microemulsion characteristics of the O/W system. F3-RPE30 exhibited a substantial increase in viscosity after incorporation of 30% RPE. This increase occurred concomitantly with the appearance of localized birefringent features observed by PLM, indicating that the rheological and optical properties of the system were both modified after RPE incorporation. F4-RPE50 also showed a marked increase in viscosity, accompanied by the appearance of localized birefringent features and larger dispersed structures observed by PLM. The concurrent increases in conductivity and viscosity indicate that RPE incorporation affected both electrical transport and flow resistance within the systems. Similar composition-dependent changes in these physicochemical properties have been reported in colloidal ion systems [56,71,73].

The Newtonian behavior observed for F3-RPE30 and F4-RPE50 may appear unexpected considering the localized optical anisotropy detected by PLM, since organized anisotropic systems are often reported to exhibit non-Newtonian, particularly pseudoplastic, behavior due to structural reorientation under shear [74]. However, localized optical anisotropy does not necessarily imply sufficient long-range organization or mechanical coupling to produce detectable non-linear rheological behavior over the investigated shear-rate range [34]. In addition, RPE constituents may influence the rheological response through interactions with the colloidal components. Phenolic compounds and flavonoids have been reported to interact with surfactant assemblies and modify interfacial properties, potentially affecting their response to shear [59,75]. It should also be noted that the rheological measurements were performed over a broad shear-rate range (0–200 s^−1^), and the very-low-shear region was not characterized at sufficiently high resolution to exclude subtle deviations from Newtonian behavior under those conditions.

Taken together, the changes in hydrodynamic diameter, electrical conductivity, viscosity, and optical behavior observed after RPE incorporation demonstrate that increasing RPE loading substantially altered the physicochemical behavior of the formulations. Notably, the localized birefringence observed in F3-RPE30 and F4-RPE50 provides evidence of optical anisotropy that was not detected in their corresponding RPE-free systems. However, the techniques employed in the present study provide indirect information on structural organization and do not allow unambiguous assignment of specific lamellar, bicontinuous, or liquid-crystalline structures, nor determination of the precise spatial localization of the birefringent features. Further structural investigations using techniques such as small-angle X-ray scattering (SAXS) and high-resolution microscopy will therefore be necessary to elucidate the nature and spatial organization of the structures associated with these physicochemical and optical changes.

### 3.5. Determination of Markers in RPE-Loaded Colloidal Systems Using UFLC-DAD-UV–Vis

The chemical composition of red propolis is distinctive due to its high isoflavone content, which distinguishes it from other types of propolis [7,76]. This composition can vary according to geographical region [5] and seasonality [8]. Some chemical compounds, however, are found more frequently, including formonetin, biochanin A, quercetin, pinocembrin, daidzein, vestitol, medicarpin, homopterocarpin and liquiritigenin [7,76,77,78,79]. Therefore, identifying chemical markers is essential for confirming the type and geographical origin of propolis, as well as evaluating the capacity and quality of incorporating RPE and its respective compounds into developed systems.

The markers (metabolites) liquiritigenin (1), daidzein (2), pinobanksin (3), isoliquiritigenin (4), formononetin (5), pinocembrin (6) and biochanin A (7) were identified and quantified by direct comparison with analytical standards, using the UFLC-DAD profile of RPE and RPE-loaded colloidal systems. The values for the retention times, maximum wavelengths and corresponding concentrations of the markers are shown in Table 4.

Although all samples were prepared to contain the same equivalent RPE concentration for chromatographic analysis, differences in the concentrations of individual markers measured in RPE and the RPE-loaded colloidal systems were observed (Table 4). All samples were subjected to the same 60-min sonication procedure prior to dilution and chromatographic analysis, as described in Section 2.9. The differences observed among formulations may reflect differences in the physicochemical properties of the markers and their interactions with the distinct colloidal environments of each formulation.

The phenolic markers evaluated in this study, including daidzein, isoliquiritigenin, formononetin, and biochanin A, are characterized by low aqueous solubility, as previously reported [80,81,82,83]. This limited aqueous solubility suggests that their incorporation into the formulations may depend on their partitioning and molecular interactions within the different colloidal microenvironments, including the oil phase and surfactant-rich interfacial regions.

However, the intermediate octanol/water partition coefficient (logP) values (2.2–3.6) and polar surface areas (66–87 Å^2^) confer an amphiphilic character that may influence their partitioning within the formulations and contribute to the observed differences in marker concentrations.

F4-RPE50 showed a distinct recovery profile for the evaluated phenolic markers. The heterogeneous physicochemical environment provided by the oil, water, and S/CoS components may offer regions of different polarity capable of accommodating amphiphilic compounds with moderate logP and intermediate Polar Surface Area (PSA) values. In this context, compounds such as liquiritigenin, daidzein, isoliquiritigenin, formononetin, biochanin A and pinobanksin exhibited higher measured concentrations than the crude extract. This behavior may reflect a balance between hydrophobic affinity and polar interaction capacity, allowing partitioning between microenvironments of different polarity, particularly oil-rich and surfactant-rich interfacial regions, which may influence the concentrations of individual markers measured under the analytical conditions employed [84].

Pinobanksin, in particular, stood out due to its high PSA value (87 Å^2^), which may favor multiple hydrogen-bonding interactions within surfactant-rich interfacial regions, potentially contributing to its higher measured concentration in F4-RPE50. In contrast, despite having logP and PSA values comparable to those of liquiritigenin (logP 2.2; PSA 66.8 Å^2^), pinocembrin (logP 2.7; PSA 66.8 Å^2^) lacks hydroxyl groups on the B ring of its flavonoid structure. This structural difference may reduce its capacity for polar interactions at the interface and favor partitioning toward more hydrophobic microenvironments, potentially contributing to its lower measured concentration in F4-RPE50.

F3-RPE30 exhibited a different marker concentration profile from F4-RPE50, particularly for pinocembrin. Considering its relatively high hydrophobicity and lower capacity for polar interactions, as discussed above, the lower measured concentration of pinocembrin may reflect preferential partitioning into hydrophobic microenvironments and differences in its accessibility within the formulation. Differences in the physicochemical environment and accessibility of colloidal regions have been reported to influence the partitioning and diffusion of hydrophobic compounds in related systems [85]. In contrast, F2-RPE12 remained optically isotropic after RPE incorporation and showed a distinct marker concentration profile, indicating that the observed differences depend not only on RPE loading but also on the physicochemical environment provided by each formulation. Overall, the differences observed among F2-RPE12, F3-RPE30, and F4-RPE50 suggest that the interplay between marker molecular properties and the distinct colloidal microenvironments of the formulations may influence their partitioning and the concentrations measured under the analytical conditions employed.

### 3.6. Total Phenolic Content and Total Flavonoid Content

The total phenolic content (TPC) and total flavonoid content (TFC) of red propolis extract (RPE) and RPE-loaded colloidal systems are shown in Table 5. RPE had a TPC of 284.6 ± 18.4 mg catechin equivalent/g of RPE and a TFC of 68.3 ± 1.0 mg quercetin equivalent/g of RPE. These values are consistent with those reported in the literature for Brazilian red propolis, which is recognised for its high isoflavone content [79,86]. After incorporation, a reduction in these parameters was observed in all three systems: F2-RPE12 showed the lowest values, F3-RPE30 showed intermediate values and F4-RPE50 showed the highest values.

While the F4-RPE50 results are consistent with the individual marker analysis, discrepancies were observed in the F2-RPE12 and F3-RPE30 systems, with overall TPC/TFC values that did not directly follow the pattern observed for the individual markers. This difference may be related to the contribution of phenolic compounds not included in the individual marker analysis, as well as to differences in the physicochemical microenvironments of the formulations, which may influence compound partitioning and accessibility. Despite the reduction compared with RPE, the RPE-loaded colloidal systems retained substantial flavonoid contents (42.1–54.6 mg QE/g of RPE), which were higher than that reported for red propolis-loaded PLA nanoparticles by Costa Silva et al. (2022) [87] (35.8 mg QE/g). Furthermore, all the samples evaluated are within the quality standard established by the Ministry of Agriculture, Livestock and Supply, which requires a minimum of 0.25% (*w/w*) flavonoids and 0.50% (*w/w*) phenolic compounds [88].

### 3.7. DPPH• Radical Scavenging Capacity Method

The DPPH• radical scavenging capacity was evaluated in RPE and in RPE-loaded colloidal systems, and the results are presented in Table 5. RPE showed an IC_50_ of 12.34 µg·mL^−1^, a value consistent with that reported in the literature for Brazilian red propolis, which generally exhibits an IC_50_ ranging from 8 to 20 µg·mL^−1^, depending on its botanical origin and the assay method employed [7,76,77,87].

After incorporation, relevant differences were observed between the systems. F2-RPE12 exhibited the lowest antioxidant activity (IC_50_ = 20.50 µg·mL^−1^), which is consistent with its low TPC and TFC values. F3-RPE30 exhibited intermediate activity (IC_50_ = 9.27 µg·mL^−1^), surpassing F2-RPE12. This may be related to the higher TPC observed for F3-RPE30 compared with F2-RPE12, despite the lower concentrations of several individual markers measured in F3-RPE30. F4-RPE50 stood out with an IC_50_ of 8.93 µg·mL^−1^—a value even lower than that of the crude extract—consistent with its higher TPC and TFC values among the RPE-loaded colloidal systems.

The lower antioxidant activity observed for F2-RPE12, despite the incorporation of phenolic and flavonoid compounds, may indicate lower accessibility of these compounds to the DPPH• radical. Their distribution and interactions with the formulation components may influence the fraction available to react with the radical. Conversely, the higher RPE loading and phenolic/flavonoid content of F3-RPE30 and F4-RPE50 may contribute to a greater fraction of antioxidant compounds available to react with the DPPH• radical under the assay conditions, consistent with their lower IC_50_ values.

It is important to note that F4-RPE50 exhibited a lower IC_50_ than the pure extract and than those reported for red propolis-loaded polymeric nanoparticles (16.10–31.30 μg·mL^−1^) [87,89]. Red propolis-loaded microcapsules have shown IC_50_ values ranging from 8.90 to 15.60 μg·mL^−1^ [90], placing F4-RPE50 (8.93 μg·mL^−1^) at the lower end of this range and supporting its high antioxidant activity.

Taken together, these results indicate that F4-RPE50 showed the most favorable phytochemical and antioxidant profile among the RPE-loaded colloidal systems, as reflected by the concentrations of the evaluated phenolic markers, its higher phenolic and flavonoid contents, and its high DPPH• radical-scavenging activity.

### 3.8. Antileishmanial Activity

**Figure 5:**
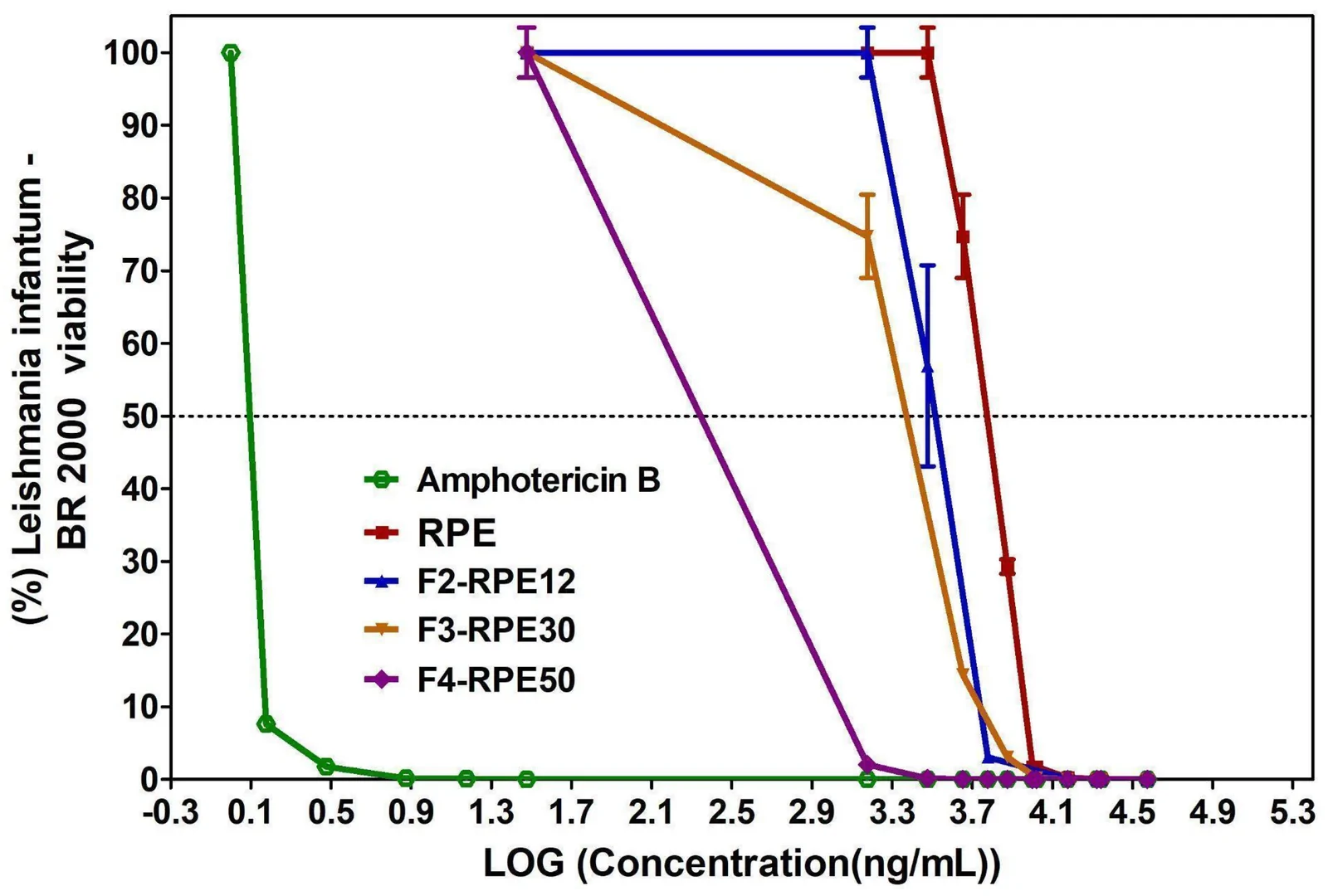
Concentration–response curves for the in vitro antileishmanial activity of RPE and RPE-loaded colloidal systems against *L. infantum* promastigotes. Amphotericin B was used as the reference drug. Parasite viability was normalized to the untreated control, and IC_50_ values were estimated by nonlinear regression.

The leishmanicidal activity of RPE and the RPE-loaded colloidal systems (F2-RPE12, F3-RPE30 and F4-RPE50) was evaluated against *L. infantum* promastigotes and expressed as IC_50_ with a 95% confidence interval (see Figure 5). Liposomal amphotericin B (AmBisome^®^, Gilead Sciences, Inc., La Verne, CA, USA), used as a therapeutic reference, had an IC_50_ value of 0.4082 ng·mL^−1^, confirming its high antiparasitic potency. Although markedly less potent than the reference drug, RPE exhibited relevant leishmanicidal activity (IC_50_ = 5.90 μg·mL^−1^), consistent with previous reports of antileishmanial activity of Brazilian red propolis [91] and supporting further investigation of its bioactive constituents.

Previous studies with Brazilian red propolis have generally reported higher IC_50_ values. The ethanolic extract of samples from Sergipe showed an IC_50_ of approximately 21.5 μg·mL^−1^ against promastigotes [9], while Regueira-Neto et al. (2018) [91] reported seasonal differences, with samples from Pernambuco collected during the rainy season showing an IC_50_ of 37.45 μg·mL^−1^ and those from Alagoas reaching 109.49 μg·mL^−1^. In another study, 20 Cuban propolis chemotypes, nine of which were classified as red propolis, exhibited IC_50_ values ranging from 3.3 to 16.1 µg·mL^−1^ against amastigotes [10]. For other types of propolis, higher IC_50_ values have also been reported. Turkish propolis exhibited IC_50_ values of 125–325 μg·mL^−1^ against promastigotes [92]; Bulgarian propolis showed values of 41.3–53.9 μg·mL^−1^; and an ethanolic propolis extract from Minas Gerais, Brazil, exhibited an IC_50_ of 49.9 μg·mL^−1^ [26]. Even the hexane fraction of propolis from the semi-arid region of Bahia, reported as the most active fraction in that study, exhibited an IC_50_ of 1.3 µg·mL^−1^ against amastigotes [93]. Overall, the IC_50_ obtained for RPE in the present study (5.90 μg·mL^−1^) compares favorably with several values reported for propolis extracts in the literature, although differences in parasite developmental stage and experimental conditions should be considered when making direct comparisons.

The leishmanicidal activity of RPE may be related, at least in part, to its distinctive phytochemical profile, which includes phenolic constituents such as liquiritigenin, isoliquiritigenin, formononetin, biochanin A, and daidzein. In silico evidence suggests that liquiritigenin may interact with the enzymatic targets LiCYP51 and TR in *Leishmania*, supporting a potential contribution of this compound to antileishmanial activity [15,27]. Furthermore, red propolis contains chalcones, which have been reported to interfere with sterol biosynthesis, leading to changes in membrane sterol composition [94]. Other studies have reported that chalcones can affect the viability of *Leishmania* species by impairing mitochondrial function and inhibiting key enzymes, including mitochondrial dehydrogenases and fumarate reductase [95].

Additionally, Monzote et al. (2011) [96] demonstrated that polyprenylated benzophenones isolated from propolis inhibited the growth of intracellular amastigotes of *L. infantum*. In a subsequent study, the same authors reported that some of these compounds specifically inhibited complexes II/III of the mitochondrial respiratory chain, leading to increased production of reactive oxygen species and subsequent parasite death, whereas others appeared to act through as-yet-uncharacterized non-mitochondrial mechanisms [97].

In addition, essential oils (EOs) generally constitute 1–3% of propolis [98]. Sena-Lopes et al. (2018) [99] reported, for the first time, the chemical composition of EOs from red propolis collected in the state of Sergipe, identifying methyl eugenol as the major constituent and α-pinene in smaller amounts. Several studies have demonstrated the leishmanicidal activity of eugenol and essential oils rich in this compound [100,101,102,103,104]. Proposed mechanisms reported in the literature include direct effects on the parasite as well as modulation of host immune responses, with upregulation of Th1-associated cytokines and downregulation of Th2-associated cytokines.

Furthermore, molecular docking studies suggest that eugenol may interact with cytokines such as TNF-α and IL-12p40, supporting a potential immunomodulatory role [105]. Eugenol derivatives have also been associated with enhanced leishmanicidal activity [106,107]. In addition, essential oils (EOs) from Tunisian propolis, which are rich in α-pinene, have been shown to exhibit activity against *L. infantum* promastigotes (IC_50_ = 3.67 μg·mL^−1^) [108], while α-pinene itself demonstrated relevant activity against promastigotes (IC_50_ = 19.7 μg·mL^−1^) [109]. The leishmanicidal properties of EOs have been mainly attributed to monoterpenes, whose lipophilic nature may facilitate their interaction with parasite membranes, potentially disrupting membrane integrity and allowing access to intracellular targets [110].

Taken together, the diverse phytochemical composition of red propolis, including isoflavonoids, chalcones, benzophenones, and potentially bioactive volatile constituents, may contribute to its leishmanicidal activity through multiple mechanisms reported in the literature, as summarized in Figure 6. Although the contribution and possible interactions among these constituents were not directly investigated in the present study, their combined presence may contribute to the observed activity of RPE.

The RPE-loaded colloidal systems exhibited progressively lower IC_50_ values than RPE alone: F2-RPE12 = 3.14 μg·mL^−1^ (1.88-fold lower), F3-RPE30 = 2.28 μg·mL^−1^ (2.59-fold lower), and F4-RPE50 = 0.56 μg·mL^−1^ (10.54-fold lower) compared with RPE alone. Among the RPE-loaded colloidal systems, F4-RPE50 was the most active, although it remained markedly less potent than the reference drug AmBisome^®^ (IC_50_ = 0.4082 ng·mL^−1^). The higher RPE loading and the physicochemical environment of F4-RPE50 may be relevant to its observed activity.

It should be noted that the corresponding RPE-free formulations (F2, F3, and F4) were not evaluated in the antileishmanial assay. Therefore, although the RPE-loaded colloidal systems exhibited lower IC_50_ values than RPE alone, the present experimental design does not allow the relative contributions of RPE, formulation excipients, and the colloidal environment to the observed activity to be distinguished. Evaluation of the corresponding RPE-free vehicles will be necessary in future studies to clarify these contributions. In addition, cytotoxicity against mammalian host cells was not evaluated in the present study; consequently, a selectivity index could not be determined. Therefore, the IC_50_ values reported here should be interpreted as evidence of in vitro antileishmanial activity rather than therapeutic selectivity. Future studies should include parallel host–cell cytotoxicity assays and determination of the selectivity index to more comprehensively assess the biological potential of these formulations.

Some red propolis delivery systems have been shown to exhibit leishmanicidal activity in the literature, albeit with higher IC_50_ values than those obtained in the present study. Polymeric nanoparticles showed an IC_50_ of approximately 31.3 µg·mL^−1^ [89], while microcapsules containing the extract exhibited IC_50_ values ranging from 8.9 to 15.6 µg·mL^−1^ [90]. In comparison, F4-RPE50 exhibited an IC_50_ of 0.56 µg·mL^−1^, supporting further investigation of its antileishmanial activity, including evaluation of host–cell cytotoxicity and selectivity.

## 4. Conclusions

RPE-loaded colloidal systems derived from microemulsion compositions were developed and exhibited distinct physicochemical, phytochemical, and biological properties. Of the formulations evaluated, F4-RPE50 stood out due to its higher phenolic and flavonoid contents, higher antioxidant activity, and the highest antileishmanial activity among the RPE-loaded colloidal systems. Importantly, physicochemical characterization showed that F4-RPE50 exhibited characteristics that were no longer consistent with those of a classical microemulsion, supporting its conservative description as an RPE-loaded colloidal system derived from a microemulsion composition. The changes in physicochemical properties observed after RPE incorporation, together with the localized birefringence detected by PLM, indicate that the extract modifies the colloidal organization of the formulations, although the specific nature of this organization requires further investigation using direct structural characterization techniques. Overall, the results demonstrate the potential of colloidal systems derived from microemulsion compositions as carriers for red propolis bioactive compounds and support further investigation of these formulations for pharmaceutical applications.

## Figures and Tables

**Table 1:** Quantitative solubility of RPE in individual oil-phase and interfacial-phase components and in S/CoS mixtures evaluated for microemulsion development.

Components	Candidates	Solubility (µg·mL^−1^)
Oil Phase	Oleic acid	7.67
	Moringa oleifera Oil	4.09
	Baru Oil	3.21
Surfactant	Tween 20	6.64
	Tween 80	9.93
Co-Surfactant (Co-S)	Ethanol	5.37
Surfactant and Co-Surfactant in Binary System or in Ternary System (Sm)	Tween 20/Ethanol (1:1)—Sm 1	9.02
	Tween 20/Ethanol (2:1)—Sm 2	8.55
	Tween 20/Ethanol (3:1)—Sm 3	16.72
	Tween 80/Ethanol (2:1)—Sm 4	7.90
	Tween20/Tween80/Ethanol (3:1:1)—Sm 5	9.66

**Table 2:** Composition, electrical conductivity, and pH of the selected RPE-free microemulsions and corresponding RPE-loaded colloidal systems.

Formulation	Oil (% *v*/*v*)	Sm (% *v*/*v*) T20:ethanol 2:1	Water (% *v*/*v*)	Conductivity (µS·cm^−1^)	pH
F2	5	45	50	92.64	3.88
F3	8	72	20	22.19	5.09
F4	9	81	10	12.61	5.42
F2-RPE12	5	45	50	259	3.99
F3-RPE30	8	72	20	71	4.34
F4-RPE50	9	81	10	28	4.10

**Table 3:** Rheological parameters (η and R^2^) and dynamic light scattering (DLS) results, including mean hydrodynamic diameter (Z-average ± SD) and polydispersity index (PDI ± SD), of the RPE-free microemulsions and RPE-loaded colloidal systems.

Formulation	η (mPa.s)	R^2^	Z-Ave ± SD (nm)	PDI ± SD
F2	43.9	0.9999	23.88 ± 0.55	0.461 ± 0.01
F3	35.7	0.9999	74.65 ± 1.05	0.445 ± 0.12
F4	10.2	0.9999	31.35 ± 1.35	0.374 ± 0.02
F2-RPE12	60.8	0.9999	180.0 ± 2.40	0.190 ± 0.01
F3-RPE30	56.9	0.9998	241.3 ± 2.52	0.279 ± 0.04
F4-RPE50	76.3	0.99996	557.0 ± 2.25	0.223 ± 0.01

**Table 4:** Determination of red propolis flavonoids and isoflavonoids by the UFLC-DAD-UV–Vis.

				Concentration (µg·mL^−1^)			
Markers	λmax (nm)	Peak No. *	RT (min)	RPE	F2-RPE12	F3-RPE30	F4-RPE50
Daidzein	249	2	12.54	2.40	2.33	1.57	3.94
Liquiritigenin	275	1	12.35	4.56	4.20	2.88	7.33
Pinobanksin	291	5	14.80	0.36	0.31	0.39	1.12
Isoliquiritigenin	366	3	17.06	4.61	4.87	3.24	9.46
*F*ormononetin	249	4	17.89	5.30	5.10	3.44	8.99
Pinocembrin	289	6	21.86	0.92	0.82	0.050	0.28
*B*iochanin A	260	7	23.10	0.12	0.19	0.31	0.66

* Peak No. corresponds to the identification number of each marker in the chromatographic profile.

**Table 5:** Phytochemical assays performed on Alagoas red propolis extract (RPE) and RPE-loaded colloidal systems.

Samples	TPC (mg CE/g of RPE ± SD)	TFC (mg QE/g of RPE ± SD)	DPPH IC_50_ (µg·mL^−1^)
RPE	284.6 ± 18.4	68.3 ± 1.0	12.34 (10.36–14.70)
F2-RPE12	202.2 ± 6.6 ^a^	42.1 ± 1.1 ^d^	20.50 (19.40–21.66)
F3-RPE30	224.6 ± 3.5 ^b^	46.3 ± 1.4 ^d^	9.27 (8.30–10.34)
F4-RPE50	236.8 ± 17.2 ^c^	54.6 ± 1.0 ^d^	8.93 (8.86–10.90)

Data are expressed as mean ± SD. TPC: one-way ANOVA followed by Bonferroni’s post hoc test (^a^ *p* < 0.001, ^b^ *p* < 0.01, and ^c^ *p* < 0.05 vs. RPE). TFC: one-way ANOVA followed by Bonferroni’s post hoc test (^d^ *p* < 0.001 vs. RPE). IC_50_ values are presented with 95% confidence intervals; nonlinear fit, R^2^ > 0.99; degrees of freedom = 11.

## Data Availability

The raw data supporting the conclusions of this article will be made available by the authors upon request.
